# Supplementary material for: Deep learning of material transport in complex neurite networks
Source: Sci Rep. 2021 May 28;11:11280. doi: 10.1038/s41598-021-90724-3 (PMC8163783; doi:10.1038/s41598-021-90724-3)
Supplement: Supplementary file 1 — Supplementary Figures. [file 41598_2021_90724_MOESM1_ESM.pdf]

Supplementary Information
Deep Learning of Material Transport in Complex Neurite Networks

Angran Li, Amir Barati Farimani and Yongjie Jessica Zhang

Supplementary Figures

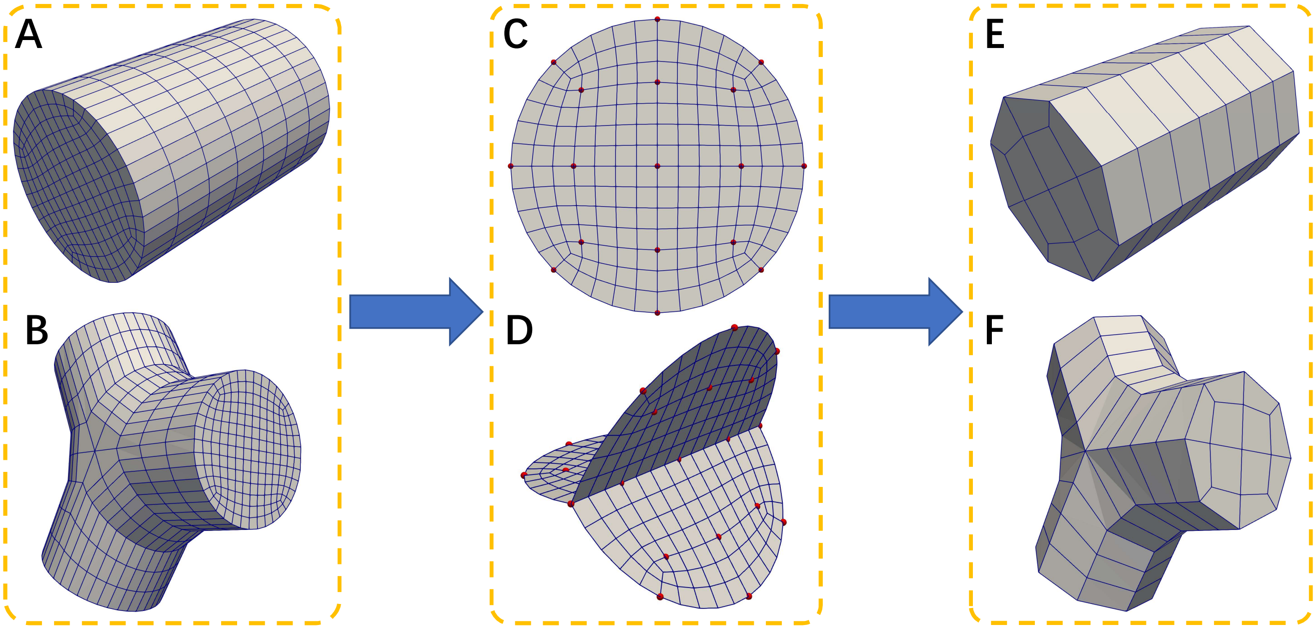

Figure S1. The graph extraction of the pipe and bifurcation structures. (A, B) The input control meshes of the pipe and the bifurcation. (C) The extraction template for circular cross sections with 17 extracted nodes labeled in red. (D) The extraction template for branch cross sections with 23 extracted nodes labeled in red. (E, F) The extracted graphs of the pipe and the bifurcation.

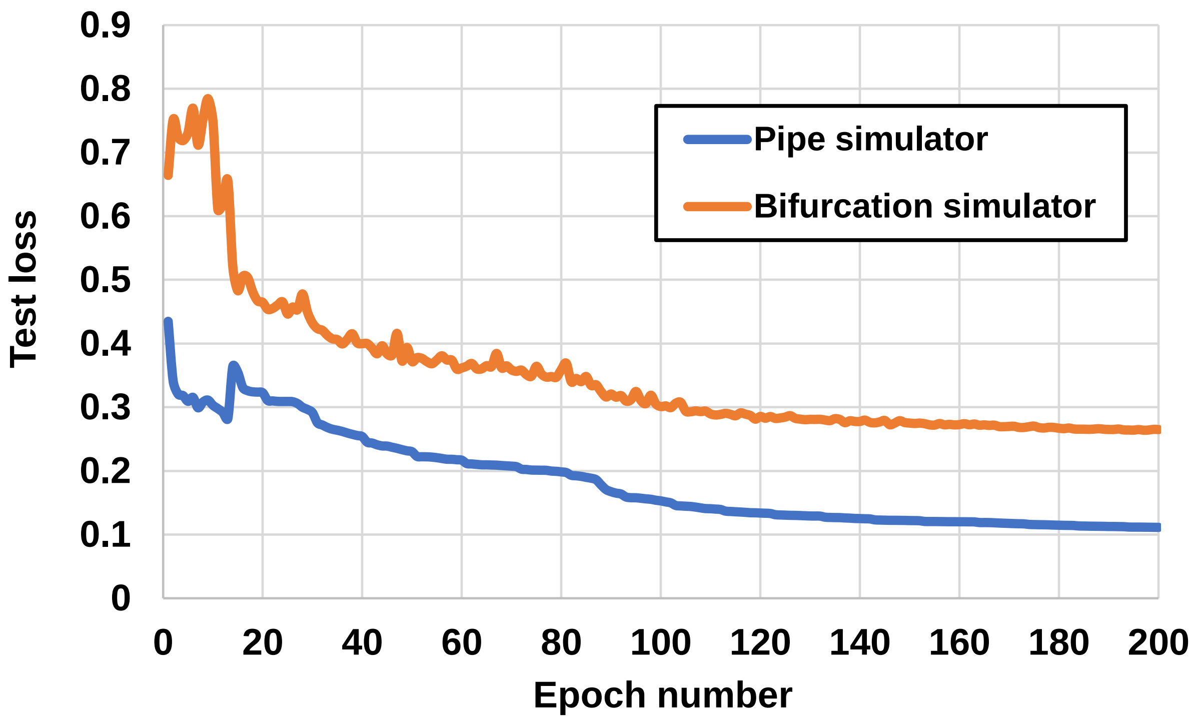

Figure S2. The test loss vs epoch curves for two GNN simulators.

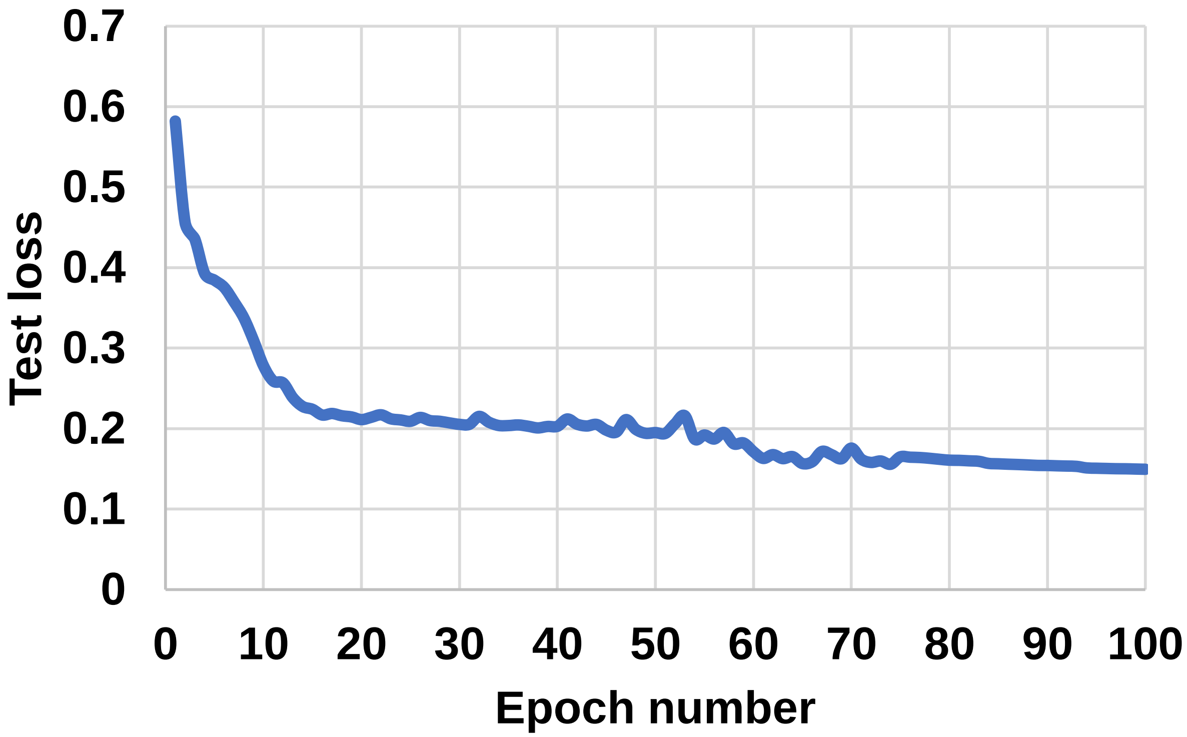

Figure S3. The test loss vs epoch curve for the GNN assembly model.

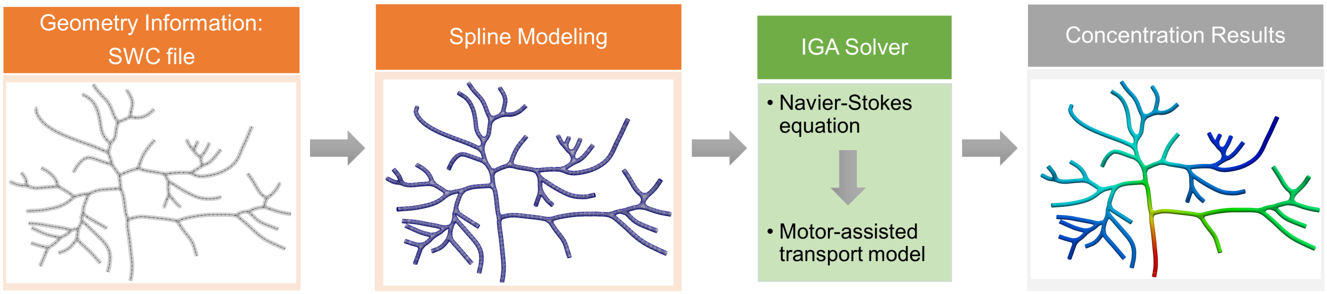

Figure S4. An overview of the IGA-based neuron material transport simulation pipeline. Given the geometry information (skeleton and diameter) of the neurite network stored in a SWC file, the spline modeling module first generates all-hexahedral control mesh and builds volumetric splines over the mesh. Then, the IGA solver takes the volumetric splines as input and computes the dynamic concentration results.

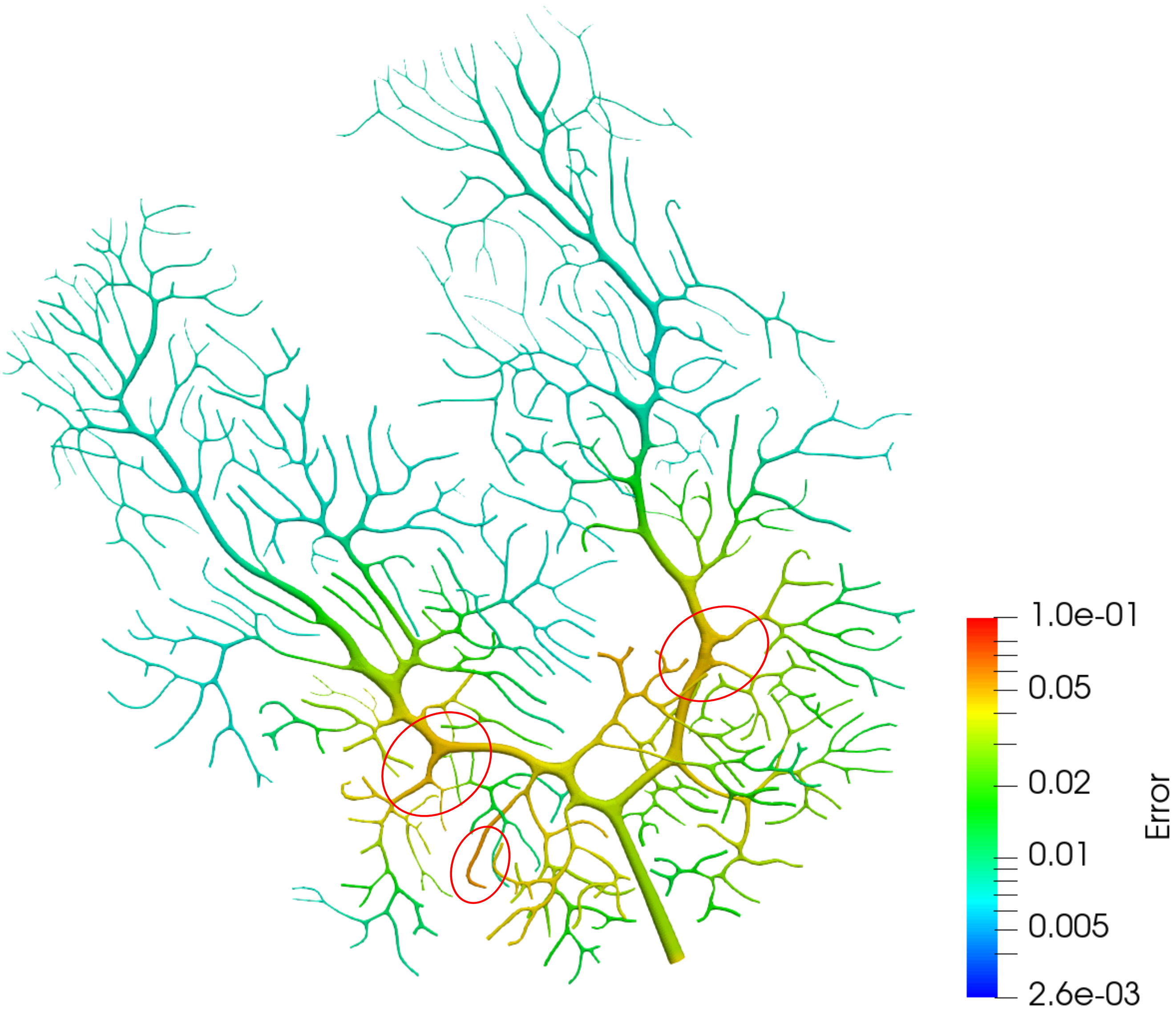

Figure S5. The prediction error of NMO_00865 using the model trained with zebrafish neuron dataset. Logarithmic scale is used to highlight the distribution pattern. The regions with higher prediction error are labeled in red circles where the geometry shows high curvature or sharp radius change.
